# The Method of Using Artificial Teeth

**Published:** 1862-02

**Authors:** 


### Editorial.

### THE METHOD OF USING ARTIFICIAL TEETH.

Patients so often ask for directions in regard to the best method of attaining familiarity with, and use of artificial teeth, especially full dentures, that we have thought a few suggestions in reference to it might not be out of place.

And first, should teeth be worn through the night as well as the day?

They should in all cases when they do not cause serious inconvenience. Familiarity with and adaptation of the denture will be much sooner attained by keeping them constantly in the mouth than by laying them out part of the time. During sleep or a time of rest, the plates become most rapidly adapted. During the day the mouth is in constant motion, and dentures are far less likely to attain a fixed position.

Again, almost every one will ask, "If I lay my teeth out at night, should I put them in water?"

If they must be put out, they should be thoroughly cleaned at once. The idea of putting a set of teeth just from the mouth into a glass of water, to thicken and ferment during the night, is rather disgusting; but then we get accustomed to almost any thing. If they are perfectly cleaned it is not necessary to put them in water.

And now, what is the best method of cleansing dentures and how often should they be cleansed. The frequency will depend upon circumstances, such as the health of the wearer, the conditions of the mouth and its secretions. Every one will be able to know by his own experience, how often his teeth should be cleansed. It should be sufficiently frequent to prevent the decomposition of any animal or vegetable substances about them; or the production of any disagreeable odor.

The *manner* of doing it will also depend upon the condition of the mouth and its secretions; the mode of construction of the denture will have much to do with it. That style of work having the fewest joints and interstices will be the easiest kept clean; of these continuous gum work stands at the head, and perhaps rubber work next; the ordinary gold and silver work being the worst in this respect. To clean the former, a good brush and clean water, with soap or chalk, will be quite sufficient; but for the latter all this and far more is required to maintain even a respectable degree of cleanliness; picks are required, and frequent boiling in some strong alkali, perhaps the lye of wood ashes is as good as any thing. This will remove animal and vegetable substances from the denture, after this, washing out perfectly with pure water: this will purify the worst cases.

Again: "*When* and *how* should I begin to eat with my teeth?"

Begin to eat with them as soon as command is obtained sufficient to keep them in place; in many instances it is well to begin before this. The facility with which artificial teeth are used for mastication is modified by several things. The tact and ability of the patient has much to do with it; some will masticate from the beginning with facility, with a set of teeth that fit no better than others, where months are required to get even a moderate degree of masticating power. Patience, perseverance, and a faith that one can do what others can will accomplish much.

The facility of accurate articulation of the teeth is lost in a great measure by those who have been long without them; the muscles become lax, and the lower jaw loses its precision of motion; in consequence of this, when an artificial denture is inserted, inaccurate occlusion of the teeth gives trouble, that is not soon, nor easily overcome. In this consists a very strong argument for the insertion of the artificial teeth immediately after the removal of the natural organs, when the patient will usually masticate well, as soon as the soreness of the gums is gone.

In regard to the mode of learning to eat, perhaps each one learns much that is peculiar to his own case.

But it may be observed as a general rule that, at first, the movement of the lower jaw should be as nearly vertical as possible, let there be little or no lateral motion in the beginning; that will be attained gradually, and in addition to the vertical motion, let the food be placed between the teeth of both sides at the same time, and thus the pressure being upon both sides of the plate will serve to fix it in place, which would not be the tendency if the pressure was all upon one side. Then begin with a vertical motion of the jaw, with food upon both sides.

Artificial dentures sometimes fail to bring out the annunciation as clearly as desirable. This in many instances is doubtless owing to the improper construction of the denture. It is, however, usually dependant upon the circumscribing of the tongue's action; when it is this alone, the difficulty is soon overcome, for the tongue will soon accommodate itself to the space in which it has to act. If the difficulty exists in the teeth, they should be changed; if it is not here, the patient should speak deliberately and without affectation, and the difficulty will soon be overcome.

In reference to wearing partial sets of teeth, the same principle should be observed as in full sets.

In cases where an attachment is made by clasps to the natural teeth, great cleanliness should be observed. The teeth to which the clasps are attached should be thoroughly cleansed two or three times a day, perhaps, immediately after each meal would not be too often. The plates of such pieces should set firmly and closely upon the gums, so as to admit of no oscillation during ordinary usage: for by such movement, the clasped teeth will often become irritated, diseased, and loosened. There is usually little or no difficulty experienced in using this kind of partial sets of teeth, especially if they are well adapted. Partial pieces, that are retained by atmospheric pressure, sometimes require considerable tact and experience by the wearer, before they become entirely familiar and useful.

Atmospheric pressure plates are almost always preferable to those attached by clasps.

